# Hantaan Virus Surveillance Targeting Small Mammals at Nightmare Range, a High Elevation Military Training Area, Gyeonggi Province, Republic of Korea

**DOI:** 10.1371/journal.pone.0118483

**Published:** 2015-04-15

**Authors:** Terry A. Klein, Heung-Chul Kim, Sung-Tae Chong, Jeong-Ah Kim, Sook-Young Lee, Won-Keun Kim, Peter V. Nunn, Jin-Won Song

**Affiliations:** 1 Force Health Protection and Preventive Medicine, 65th Medical Brigade/US Army MEDDAC-Korea, Unit 15281, APO AP 96205–528, United States of America; 2 5th Medical Detachment, 168^th^ Multifunctional Medical Battalion, 65th Medical Brigade, Unit 15247, APO AP 96205–5247, United States of America; 3 Department of Microbiology, College of Medicine, Institute of Biomedical Science & Food Safety, Korea University, 126–1, 5-ga, Anam-dong, Seongbuk-gu, Seoul, 136–705, Republic of Korea; Icahn School of Medicine at Mount Sinai, UNITED STATES

## Abstract

Rodent-borne disease surveillance was conducted at Nightmare Range (NM-R), near the demilitarized zone in northeast Gyeonggi Province, Republic of Korea, to identify hemorrhagic fever with renal syndrome (HFRS) risks for a mountainous high-elevation (500 m) military training site. Monthly surveys were conducted from January 2008-December 2009. A total of 1,720 small mammals were captured belonging to the Orders Rodentia [Families, Sciuridae (1 species) and Muridae (7 species)] and Soricomorpha [Family, Soricidae (1species)]. *Apodemus agrarius*, the primary reservoir for Hantaan virus (HTNV), accounted for 89.9% (1,546) of all small mammals captured, followed by *Myodes regulus* (4.0%), *Crocidura lasiura *(3.9%), *Micromys minutus* (1.4%), *Mus musculus* (0.3%), *Microtus fortis *(0.2%), *Apodemus peninsulae* (0.2%), *Tamias sibiricus* (0.1%), and *Rattus norvegicus *(<0.1%). Three species were antibody-positive (Ab+) for hantaviruses: *A*. *agrarius* (8.2%), *M*. *minutus* (4.2%), and *C*. *lasiura* (1.5%). HTNV specific RNA was detected in 93/127 Ab+ *A*. *agrarius*, while Imjin virus specific RNA was detected in 1/1 Ab+ *C*. *lasiura*. Overall, hantavirus Ab+ rates for *A*. *agrarius *increased with weight (age) and were significantly higher among males (10.9%) than females (5.1%) (P<0.0001). High *A*. *agrarius *gravid rates during the fall (August-September) were associated with peak numbers of HFRS cases in Korea that followed high gravid rates. From 79 RT-PCR positive *A*. *agrarius*, 12 HTNV RNA samples were sequenced and compared phylogenetically based on a 320 nt sequence from the G_C_ glycoprotein-encoding M segment. These results demonstrate that the HTNV isolates from NM-R are distinctly separated from HTNV isolated from the People’s Republic of China. These studies provide for improved disease risk assessments that identify military activities, rodent HTNV rates, and other factors associated with the transmission of hantaviruses during field training exercises.

## Introduction

Hantaan virus (HTNV), an etiologic agent of hemorrhagic fever with renal syndrome (HFRS), poses a serious health threat to military personnel training in field environments due to its mean duration of illness from the onset of symptoms to complete recovery, overall morbidity, and a mortality rate of 5–10% in the presence of good medical management [1–3]. HTNV transmission risks are especially high near the demilitarized zone (DMZ) in the Republic of Korea (ROK) where rodent serological HTNV antibody positive (Ab+) rates were observed to be >60% during some survey periods [4–6]. Hantavirus surveillance has previously been reported for military training sites at elevations <50 m, which included host habitat characterization, seasonal small mammal population abundance and hantavirus Ab+ rates, and military activities that promoted increased infection risks [4–8]. However, hantavirus surveillance has not been conducted for high elevation (>500 m) military training sites, e.g., Nightmare Range (NM-R). This report focuses on epidemiological parameters that identify rodent hantavirus Ab+ rates and biological (e.g., seasonal gravid rates) and environmental conditions that are conducive for transmission of HTNV at NM-R. These data assist in the development of plans and decision making for instituting HFRS risk reduction strategies for US and ROK military personnel conducting military training operations, as well as civilian populations residing, working, or conducting recreational activities in mountainous regions.

## Materials and Methods

### Biosafety and Ethical Statement

All trapping of small mammals was approved by US Forces Korea (USFK) in accordance with USFK Regulation 40–1 “Prevention, Surveillance, and Treatment of Hemorrhagic Fever with Renal Syndrome” at US Military Installations and US and ROK Operated Military Training Sites [9]. Standard procedures were followed for the collection and transportation of specimens to minimize hazards from potentially infected rodents as described by Mills et al. (1995) [10] and all personnel processing rodents at the Korea University laboratory were vaccinated using a Korean-approved Hantavirus vaccine (Hantavax) [11]. Small mammals were euthanized the same day of capture by cardiac puncture under isoflurane anesthesia in strict accordance with the Korea University Institutional Animal Care and Use Committee (KUIACUC, #2010–212) protocol approved for this study.

### Site Description

**Fig 1:**
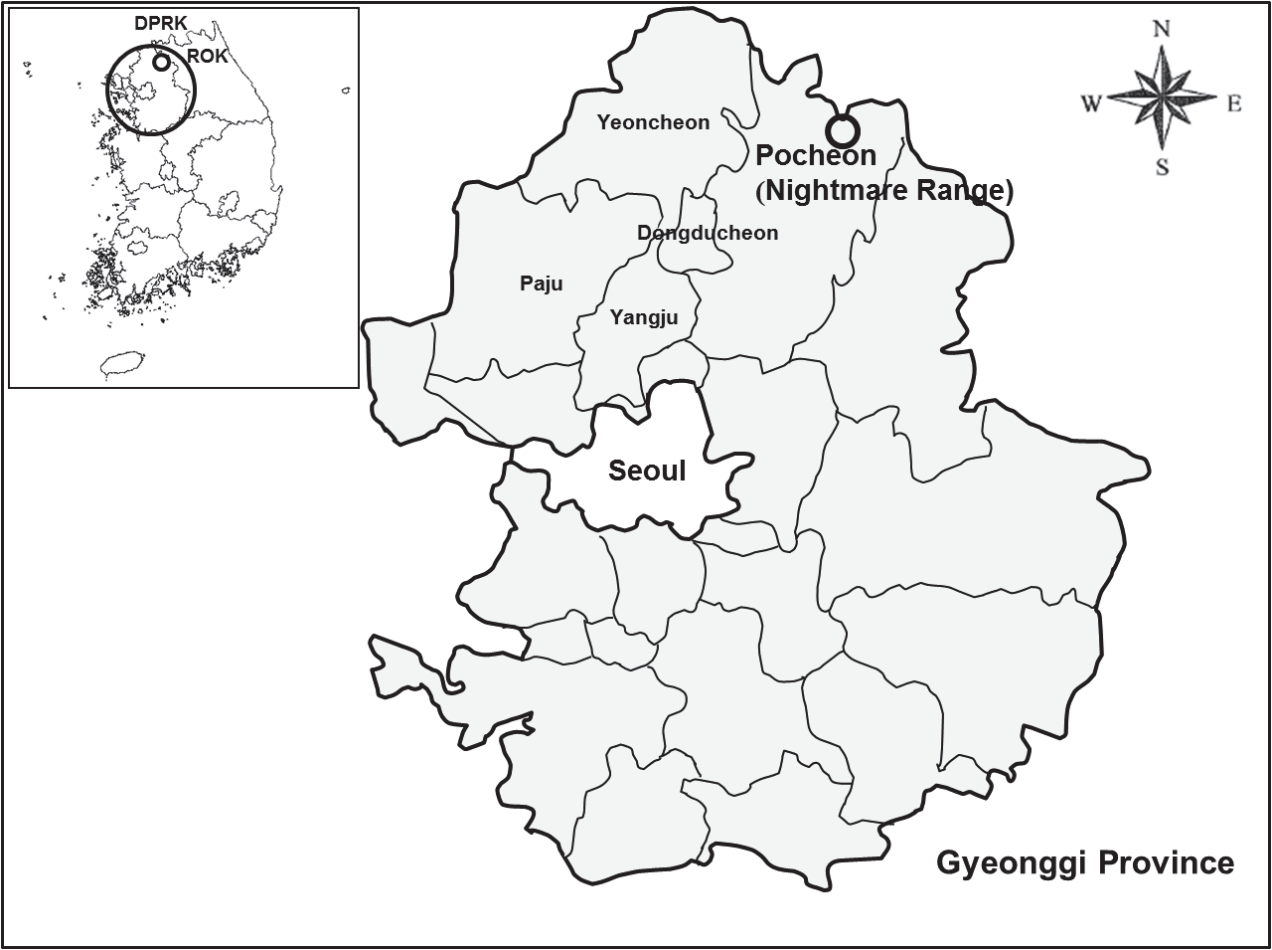
Nightmare Range, Gyeonggi Province, where rodent-borne disease surveillance was conducted monthly from January 2008-December 2009.

NM-R is a high elevation (500 m) ROK operated military training site in northern Gyeonggi province near the DMZ (Fig 1). It is characterized by a central modernized mechanized track/ wheeled firing range with short cut vegetation (not surveyed), a cantonment area consisting of wooden barracks and cut grasses bordered by unmanaged grasses/ herbaceous vegetation and mixed forests, and narrow stream valleys. Rutted dirt roads intersect narrow stream valleys to the boundary of the range and were bordered by moderate to steep hillsides with tall grasses/ herbaceous vegetation, and shrubs bordered by mixed forests to the hilltops. Troop maneuver and staging areas and mortar firing ranges, with limited vegetation at their centers, were transected by rutted dirt roads and bounded by unmanaged lands characterized by short (0.5 m) to tall grasses (>2 m) and mixes of grasses and herbaceous vegetation [e.g., giant ragweed (*Ambrosia* spp.) and *Artemisia* spp.] that provided food, cover, and harborage for small mammals. Grasses provided a food source and ground cover for the small mammals during all seasons, whereas those areas with higher proportions of herbaceous vegetation provided limited ground cover and protection for small mammals during the winter and early spring seasons. Kudzu (*Humulus japonica*), a crawling vegetation often abundant at lower elevation training areas (unmanaged lands) that provides cover for small mammals during all seasons [8],was limited.

### Small Mammal Trapping

Trapping was conducted monthly from January 2008-December 2009 among tall grass and herbaceous vegetation habitats in close proximity to the primary mechanized and mortar ranges, areas of troop and vehicle assembly and movement, and other areas that did not interfere with military training activities. Trapping was not conducted within the mechanized firing range due to entry restrictions. Collapsible live-capture Sherman traps (7.7 x 9 x 23 cm; H.B. Sherman, Tallahassee, FL) were set in grasses and herbaceous vegetation at 4–5 m intervals (25–50 traps/ trap line) that provided shade during the late afternoon over a 2–3 day period and were picked up early the following morning to avoid overheating of the captured animals during the summer period. During the late fall through spring when temperatures fell below 18°C, 3 non-absorbent cotton balls were placed in the trap to retain body heat. The following morning after each trap night, the traps were picked up and those positive for small mammals were sequentially numbered, placed in a secure container (maximum capacity 38 traps), and transported to Korea University. The small mammals were euthanized (same day), identified to species using morphological techniques, sexed, weighed, and then tissues (spleen, lung, and kidney) removed and stored at -70°C until used in this and other studies [4–6].

### Serologic and RT-PCR Test for Hantaviruses

Small mammal sera were diluted 1:16 in phosphate buffered saline and examined for IgG antibodies against HTN, Seoul (SEO), Prospect Hill (PH), and Imjin (MJN) viruses by indirect immunofluorescent antibody techniques (IFAT) [12–15]. Lung and spleen tissues from hantavirus Ab+ rodents (from sera) were initially assayed for antigens against hantaviruses by IFAT. Lung tissues of hantavirus Ab+ rodents were used for the amplification of the HTN viral gene by RT-PCR that amplified a portion of the G_C_ glycoprotein-encoding M segment. Total hantavirus RNA, extracted from lung tissues of Ab+ rodents and/ or infected Vero cells using the RNA-Bee Kit (TEL-TEST Inc., Friendswood, TX), was amplified by RT-PCR and reverse transcribed using the Superscript II RNase H-reverse transcriptase kit (Invitrogen, Carlsbad, CA) according to the manufacturer’s instructions. Nested-PCR using the primers: outer primer set, 5′-TGGGCTGCAAGTGC-3′, 5′-ACATGCTGTACAGCCTGTGCC-3′; inner primer set, 5′-TGGGCTGCAAGTGCATCAGAG-3′, 5′-ATGGATTACAACCCCAGCTCG-3′; were then used to amplify a 373-nucleotide (nt) region of the hantavirus G_C_ glycoprotein-encoding M segment [16–18]. Amplified products were size fractionated by electrophoresis on 1.5% agarose gels containing ethidium bromide (0.5 mg/mL). PCR products were cloned using the PST Blue-I vector (Novagen, Dormstadt, Germany), and plasmid DNA purified using the QIAprepSpinMiniprep kit (QIAGEN Inc., Chatsworth, CA). DNA sequencing was performed in both directions from at least three clones of each PCR product using the Big-Dye Terminator v3.1 cycle sequencing kit (Applied Biosystems, Foster City, CA) on an automated sequencer (**Model 3730**, Applied Biosystems, Foster City, CA).

### Genetic and Phylogenetic Analyses

Alignment and comparison of partial M segment sequences of HTNV strains amplified from *A*. *agrarius* captured at NM-R with previously published hantavirus sequences were facilitated using the Clustal W method (Lasergene program version 5, DNASTAR Inc. Madison, WI). The phylogenetic tree was generated by the maximum likelihood (ML) method (Molecular Evolutionary Genetics Analysis, 6.0). Genetic distances were computed by MEGA 6.0, and topologies were evaluated by bootstrap analysis of 1,000 iterations [19].

## Results

### Small Mammal Collections

A total of 1,720 small mammals belonging to the Orders Rodentia [Families, Sciuridae (1 species) and Muridae (7 species)] and Soricomorpha [Family, Soricidae (1 species)], were captured over 6,525 trap nights from January 2008-December 2009, with an overall trap rate of 26.4% (Table 1). *Apodemus agrarius* (striped field mouse), the primary reservoir for HTNV, accounted for 89.9% (1,546) of all small mammals captured, followed by *Myodes regulus* (Royal or Korean red-backed vole, 4.0%, 68), *Crocidura lasiura* (Ussuri white-toothed shrew, 3.9%, 67), *Micromys minutus* (harvest mouse, 1.4%, 24), *Mus musculus* (house mouse, 0.3%, 6), *Microtus fortis* (reed vole, 0.2%, 3), *Apodemus peninsulae* (Korean field mouse, 0.2%, 3), *Tamias sibiricus* (Siberian chipmunk, 0.1%, 2), and *Rattus norvegicus* (Norway rat, <0.1%, 1).

**Table 1:** Small mammals seropositive (%) for hantaviruses and positive (%) for Hantaan virus by reverse transcriptase-polymerase chain reaction (rt-pcr) of lung tissues at Nightmare Range from January 2008-December 2009.

Species	Ab+ rate (%)	RT-PCR positive (%)
*Apodemus agrarius*	127/1,546 (8.2)	93/127 (73.2)
*Apodemus peninsulae*	0/3	n/d^1^
*Micromys minutus*	1/24 (4.2)	0/1 (0.0)
*Microtus fortis*	0/3	n/d
*Mus musculus*	0/6	n/d
*Myodes regulus*	1/68 (1.5)	0/1 (0.0)
*Rattus norvegicus*	0/1	n/d
*Tamias sibiricus*	0/2	n/d
*Crocidura lasiura*	1/67 (1.5)	1/1 (100)
TOTAL	130/1,720 (7.6)	94/130 (72.3)

^1^n/d = not done.

**Fig 2:**
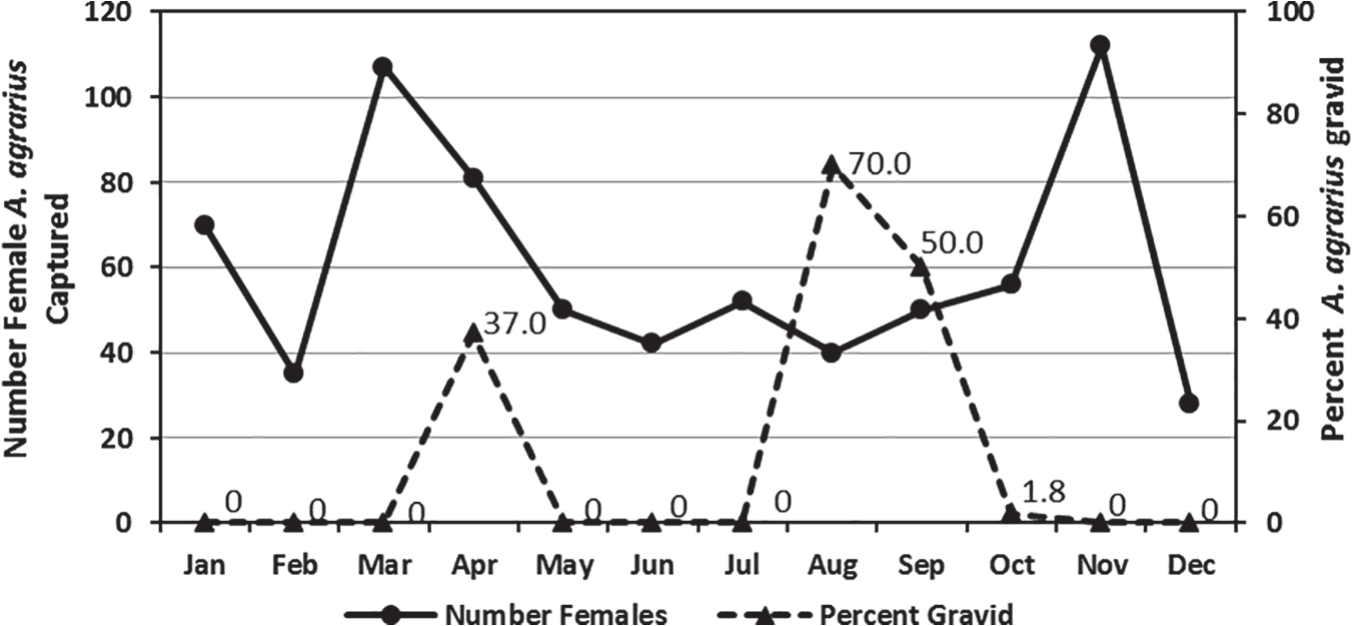
Number of *Apodemus agrarius* females captured monthly and percent gravid at Nightmare Range from January 2008-December 2009.

Overall monthly capture rates for *A*. *agrarius* were variable, ranging from 13.3% to 39.7% (mean 23.5%) (Fig 2). Gravid females were observed only during April (37.0%), and then again during August (70.0%), September (50.0%), and October (1.8%) (Fig 2). Overall gravid rates were significantly higher (χ^2^ = 4.991, df = 1, P = 0.025) during August and September (62.2%) than during April (37.0%).

**Fig 3:**
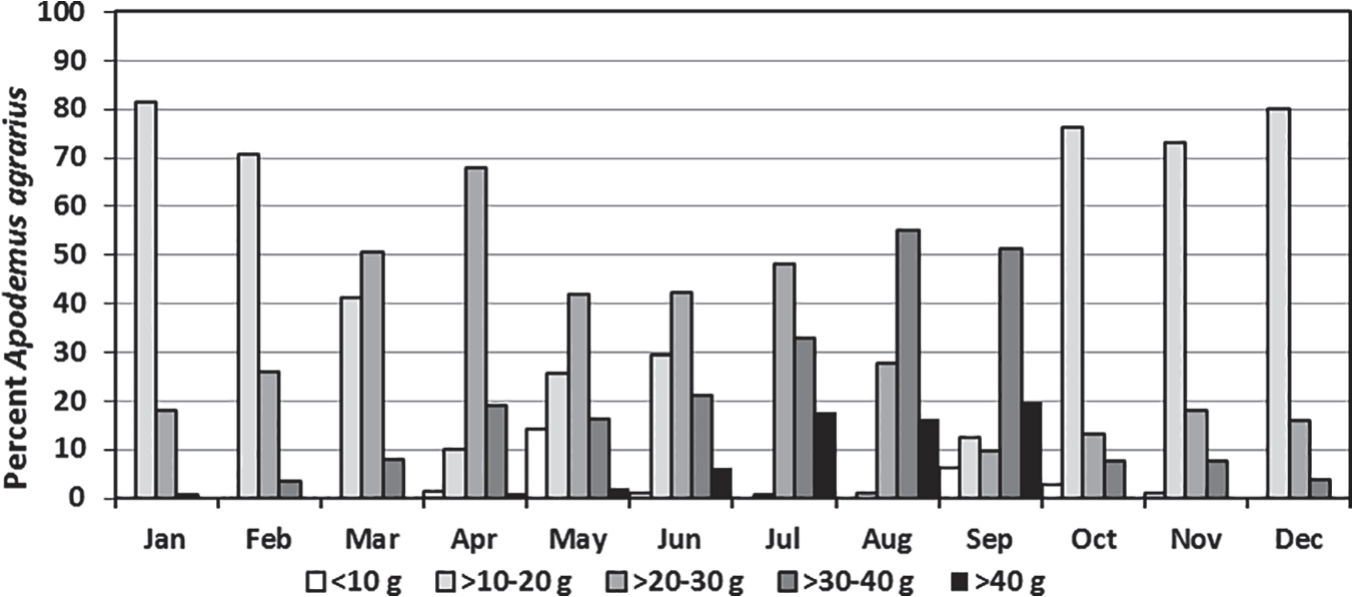
Monthly percent of *A*. *agrarius* captured at Nightmare Range^1^, by weight, from January 2008-December 2009. ^1^ One each of *Micromys minutus* and *Myodes regulus* were seropositive for hantaviruses during January 2009 when the hantavirus seropositive rate for *Apodemus agrarius* was 5.1%. One *Crocidura lasiura* was seropositive for Imjin virus during June.

Few *A*. *agrarius* weighed ≤10 g (1.9%) or >40 g (4.6%), with most weighing >10–20 g (40.8%), followed by >20–30 g (34.8%) and >30–40 g (17.9%). From January through April, the proportion of *A*. *agrarius* weighing ≤20 g declined from a high of 81.3% to 11.7%, while the proportion weighing >20g increased from a low of 18.7% to 88.3% (Fig 3). The proportion of *A*. *agrarius* weighing ≥20 g decreased to 60.2% in June following the moderate gravid rate observed in April, and then increased to 99.1% by August. Following the high fall gravid rates observed in August and September, the proportion of *A*. *agrarius* weighing ≤20 g increased in September (18.9%) and remained high from October to January the following year (74.2% to 81.3%) before declining from 70.6% (February) to 0.9% (July). The highest proportions of *A*. *agrarius* weighing ≤10 g were observed in May (14.3%) and September (6.3%), following high gravid rates during the preceding months.

### Serologic Studies

**Fig 4:**
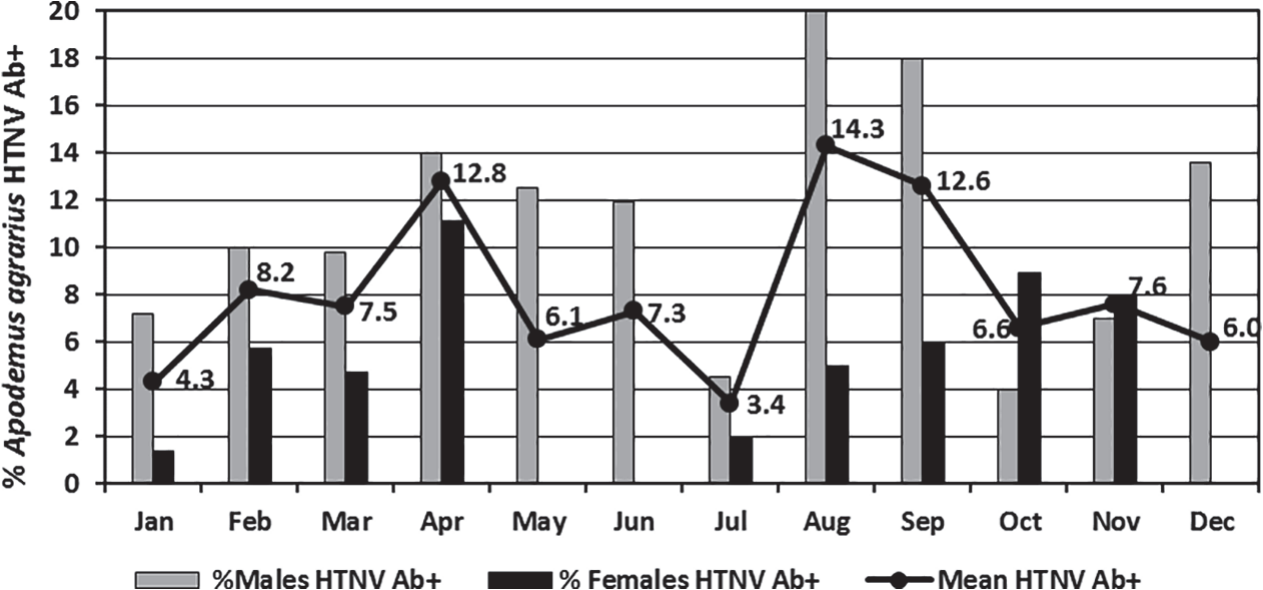
Monthly percent of male, female, and mean (line) A. **agrarius seropositive for hantaviruses from January 2008-December 2009.** A total of three, one each *Micromys minutus*, *Myodes regulus*, and *Crocidura lasiura*, were seropositive for hantaviruses (not included).

**Fig 5:**
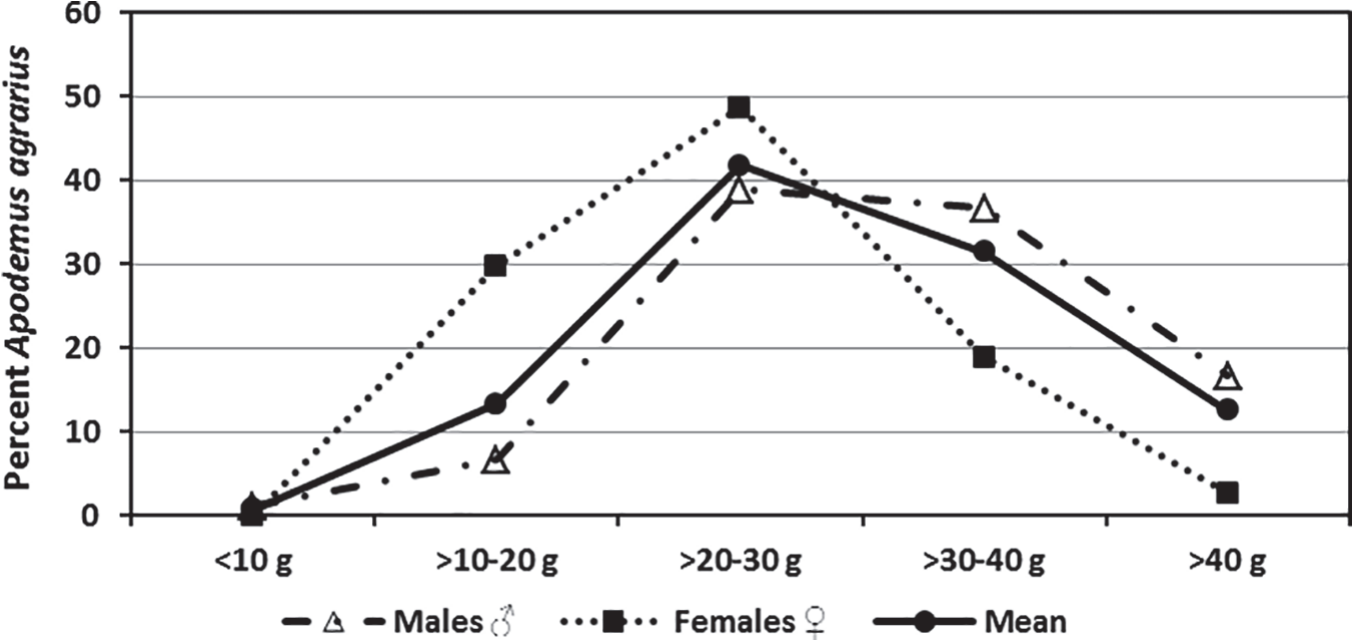
Overall proportion *A*. *agrarius* Hantavirus Ab+ for each weight category at Nightmare Range, January 2008-December 2009.

**Fig 6:**
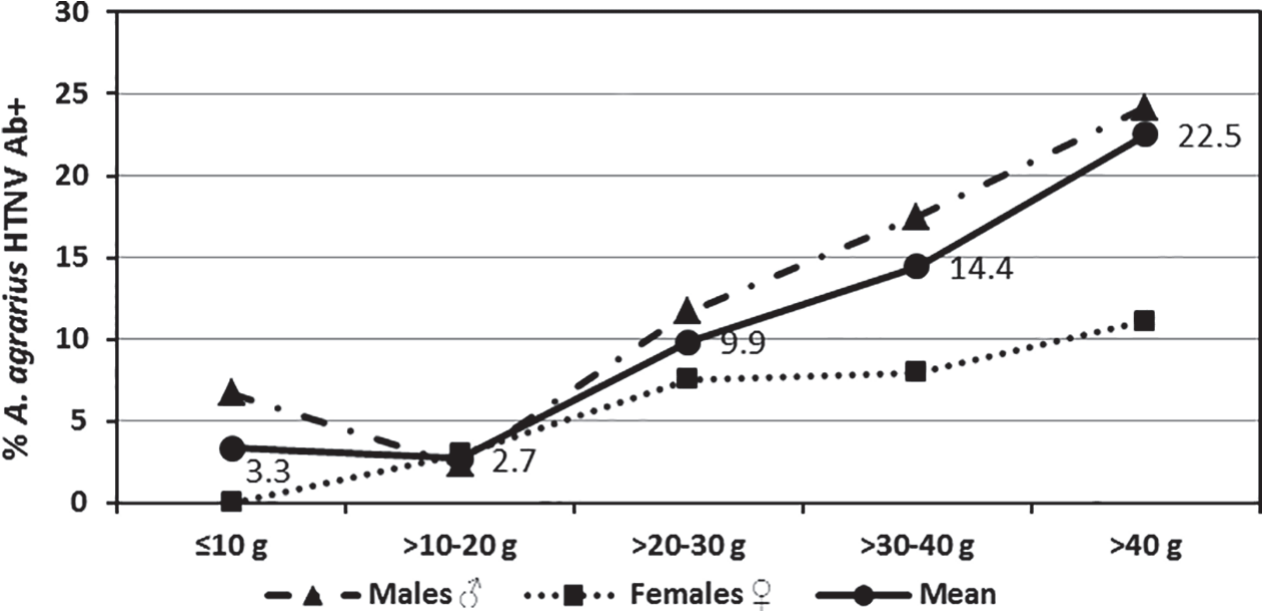
Proportion of male and female *A*. *agrarius*, by weight category, that were seropositive for hantaviruses, Nightmare Range, January 2008-December 2009.

Of the nine species of small mammals captured, IgG antibodies against hantaviruses were detected by IFAT only in *A*. *agrarius* (127/1,546; 8.2%), *M*. *minutus* (1/24; 4.2%), *M*. *regulus* (1/68; 1.5%), and *C*. *lasiura* (1/67; 1.5%) (Table 1). Of the 127 hantavirus Ab+ *A*. *agrarius*, 73.2% were confirmed HTNV positive by RT-PCR, while HTNV was not detected in tissues of hantavirus Ab+ *M*. *minutus*, *M*. *regulus*, or *C*. *lasiura*. MJNV, a newly described hantavirus from soricomorphs [14], was detected in 1/67 (1.5%) *C*. *lasiura*. Overall *A*. *agrarius* hantavirus Ab+ rates ranged from 2.1–14.3% for different trapping periods (Fig 4). The overall monthly hantavirus Ab+ rates, while variable, were significantly higher for *A*. *agrarius* males (10.9%) when compared to females (5.1%) (χ^2^ = 17.279, df = 1, P<0.001). Hantavirus Ab+ rates were especially high in male populations during August (20.0%) and September (18.0%), when there were observed high reproductive rates and subsequent to the primary mating season in July, while rates were relatively low among females during the same periods (5.0% and 6.0%, respectively). Of the total number of *A*. *agrarius* captured, the highest proportion of hantavirus Ab+ specimens weighed >20–30 g for both sexes (Fig 5). While the proportion of hantavirus Ab+ females exceeded the mean for those weighing >10–30 g, males exceeded the mean for those weighing >30 g. In general, as weight increased, the proportion of hantavirus Ab+ male and female *A*. *agrarius* within each weight category increased (Table 2 and Fig 6).

**Table 2:** Seasonal and total number of *A*. *agrarius* Ab+ for HTNV/ number captured by weight category (%) at Nightmare Range from 2008–2009.

Trapping Season	Weight Class (g)					TOTAL
	≤10	>10–20	>20–30	>30–40	>40	
Winter	0/0	7/272	18/168	6/23	0/0	31/463
(Jan-Mar)	(0.0)	(2.6)	(10.7)	(26.1)	(0.0)	(6.7)
Spring	0/18	2/76	20/215	13/75	5/11	40/395
(Apr-Jun)	(0.0)	(2.6)	(9.3)	(17.3)	(45.5)	(10.1)
Summer	0/7	0/16	5/97	15/154	11/60	31/334
(Jul-Sep)	(0.0)	(0.0)	(5.2)	(9.7)	(18.3)	(9.3)
Fall	1/5	8/266	10/58	6/25	0/0	25/354
(Oct-Dec)	(20.0)	(3.0)	(17.3)	(24.0)	(0.0)	(7.1)
TOTAL	1/30	17/630	53/538	40/277	16/77	127/1,546
% Positive	(3.3)	(2.7)	(9.9)	(14.4)	(20.8)	(8.2)

### Sequence Analysis

**Fig 7:**
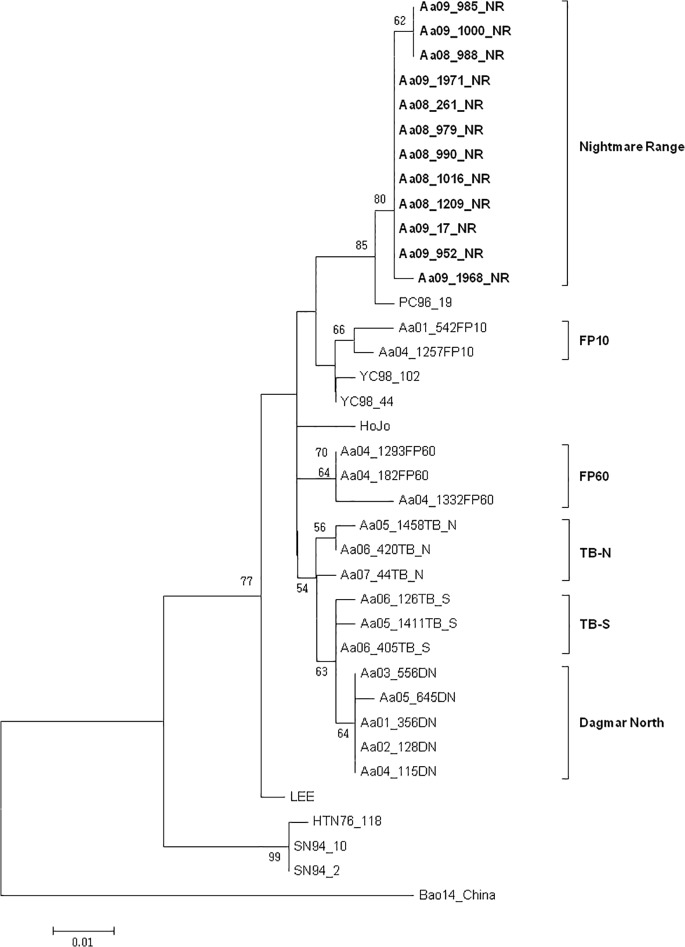
Maximum likelihood method based on a 320-nt region of the G_C_ glycoprotein-encoding M segment of the HTNV amplified from 12 *A*. *agrarius* captured at Nightmare Range (GenBank accession numbers; KM279662-KM279673). PC stands for Pocheon, and YC stands for Yeoncheon. HTNV 76–118, Lee, Hojo, and Bao 14 are included for comparison. GenBank accession numbers for HTN 76–118, HTN Lee, HTN HoJo, and HTN Bao14 are M14627, D00377, D00376, and AB127995, respectively. Branch lengths are proportional to the number of nucleotide substitutions, while vertical distances are for clarity only. The numbers at each node are bootstrap probabilities (expressed as percentages), as determined for 1000 iterations by MEGA6.0.

The genetic diversity and phylogenetic relationships among Korean strains of HTNV were determined for isolates obtained from the lung tissue of hantavirus Ab+ rodents at NM-R (Fig 7). Partial sequencing of the G_C_ glycoprotein-encoding M segment from HTNV isolates yielded a 373 nt fragment and 320 nt length, which excluded primer sequences trimmed to match the lengths of the end sequences, was used for analysis. Twelve of the 79 HTNV isolates were submitted to GenBank (Accession numbers; KM279662-KM279673) and can be accessed at: http://www.ncbi.nlm.nih.gov/nuccore/KM279662 to http://www.ncbi.nlm.nih.gov/nuccore/KM279673 (Fig 7). The partial M segment sequence (coordinates 1,993 to 2,312) of 79 HTNV RNA samples from NM-R correspond to these reference numbers: Aa08-261, Aa08-279, Aa08-285, Aa08-296, Aa08-457, Aa08-464, Aa08-472, Aa08-476, Aa08-510, Aa08-979, Aa08-984, Aa08-988, Aa08-990, Aa08-1016, Aa08-1017, Aa08-1040, Aa08-1041, Aa08-1178, Aa08-1203, Aa08-1209, Aa08-1212, Aa08-1215, Aa09-17, Aa09-216, Aa09-250, Aa09-261, Aa09-354, Aa09-369, Aa09-372, Aa09-407, Aa09-411, Aa09-571, Aa09-621, Aa09-646, Aa09-652, Aa09-766, Aa09-828, Aa09-939, Aa09-948, Aa09-949, Aa09-950, Aa09-952, Aa09-985, Aa09-1000, Aa09-1043, Aa09-1221, Aa09-1224, Aa09-1376, Aa09-1377, Aa09-1391, Aa09-1399, Aa09-1401, Aa09-1421, Aa09-1426, Aa09-1436, Aa09-1438, Aa09-1439, Aa09-1441, Aa09-1442, Aa09-1446, Aa09-1448, Aa09-1454, Aa09-1510, Aa09-1512, Aa09-1530, Aa09-1539, Aa09-1614, Aa09-1615, Aa09-1616, Aa09-1641, Aa09-1642, Aa09-1647, Aa09-1667, Aa09-1763, Aa09-1828, Aa09-1831, Aa09-1968, Aa09-1971, Aa09-1988). The nucleotide similarity and amino acid identity of the 79 HTNV RNA samples from NM-R varied from 0–3.1% and 0–2.8%, respectively.

### Phylogenetic Analysis

A Maximum Likelihood method, based on the 320-nt region of the G_C_ glycoprotein-encoding M segment of HTNV, demonstrated that HTNV isolates from *A*. *agrarius* from NM-R in the ROK aligned to form a monophyletic group, as supported by a high bootstrap probability (100%) (Fig 7).These data indicate that the ROK HTNVs represent a geographic-specific clustering based on the capture sites in Gyeonggi province, Korea and are evolutionarily distinct from HTNV from the People’s Republic of China.

Phylogenetic analysis using the Maximum likelihood method indicated that HTNV isolates from NM-R Range formed a cluster with PC96-19, originated from Pocheon, ROK, as supported by bootstrap analysis of 1,000 iterations (Fig 7).

## Discussion

Nearly all hantavirus infections among US military personnel from 1986–2013 (74/75) have been attributed to exposure while conducting military exercises at US and ROK operated military training sites near the DMZ [1,3, Klein personal communication],with only one case (SEOV) attributed to transmission at a US military installation [20]. As a result, a rodent-borne disease surveillance program was initiated in 2000 to access seasonal and annual HFRS risk factors associated with rodent bionomics and population densities, hantavirus Ab+ positive rates, and distribution of Ab+ rodents, in addition to environmental and human risk factors associated with military training activities.

### Rodent Population Abundance and Bionomics

*Apodemus agrarius* is the most commonly collected small mammal in rural environments [21–23]. Similar to this survey and other multi-year surveys at US and ROK operated military training sites at lower elevations (<50 m) near the DMZ, *A*. *agrarius* accounted for 88.5–96.1% of all small mammals captured [4,7–8]. *Apodemus agrarius* is most often associated with unmanaged lands characterized by abundant grasses/ herbaceous vegetation with capture rates from 20–60%. Lower capture rates (10–20%) were observed for grassy margins of earthen ditches that border dirt, gravel, and hardened roads, while capture rates for rice paddy banks with limited space, ground cover, and frequently cut grasses were <10% [8]. At NM-R, *A*. *agrarius* was similarly associated with tall grass habitats bordering hardened (asphalt/ concrete), gravel, and rutted dirt roads, mortar ranges, and troop maneuver/ assembly areas. The presence of large numbers of *A*. *agrarius* associated with these habitats, combined with high HTNV infection rates, vehicle and troop movement, and practice fires, increases the potential exposure and transmission resulting from aerosolized HTNV contaminated excreta during vehicle and troop movement, practice fires, and building maintenance (e.g., dry sweeping) in rodent infested buildings.

*Apodemus peninsulae*, the primary reservoir for Soochong virus, is associated with mountainous environments >500 m [24–25]. During this survey *A*. *peninsulae* only accounted for 0.2% of all small mammals collected, while none were collected in previous surveys for military training sites at elevations of <50 m [4,7–8]. A higher proportion of *Myodes regulus* (4.0%), the primary reservoir of Muju virus, was captured when compared to training sites at lower elevations (range <0.1–0.5%) [4,7–8]. While one *M*. *regulus* was serologically positive for hantavirus, Muju virus was not detected by PCR. *Rattus norvegicus*, the primary reservoir for Seoul virus and commonly collected in urban environments, is infrequently encountered at field training sites [5,20,26]. Although there was evidence of a rat infestation at the food storage and preparation building, only one *R*. *norvegicus* was captured. The low capture rate may have been, in part, due to the small size of the Sherman traps that were selected for capturing *A*. *agrarius*, the target rodent.

While gravid females were observed from March-December at lower elevations, gravid females were only observed at NM-R during April (37.0%) and then again in August-September (70.0% and 50%, respectively) that carried over into early October (1.8%). These high reproductive periods precede increased numbers of HFRS cases reported by the Korea Centers for Disease Control and Prevention [5–8,27]. Reproductive periods were reflected in the population age, indicating that *A*. *agrarius* live for approximately one year, with the majority of the older population replaced during the winter season [7–8,28]. A secondary reproductive peak (April-May) occurs when there is the emergence of new growth vegetation and primary habitats are expanding, which results in low-moderate intraspecies competition for suitable habitats. As herbaceous vegetation dies in the late fall, available habitat and ground cover is reduced. The combination of 1) mating activities (territorial disputes leading to transmission of hantaviruses through biting) that precede high gravid rates in the fall, 2) high gravid rates in August-September, 3) infusion and movement of large numbers of naïve young rodents in September-October,4) and young populations seeking suitable winter habitats greatly increases the competition for space and potential for wounding, transmission of HTNV, and increased proportion of acute infections among young naïve mice [29–30]. The overall higher hantavirus Ab+ rates observed during the fall indicates that the infusion of young naïve mice in late August-September result in increased proportions of acute HTNV infections and potential for human transmission, which was reflected in the observed higher numbers of HFRS cases reported from late September-December [27,31–33]. Territories are established by November-December, reducing conflict, wounding (rodent-to-rodent HTNV transmission), which results in fewer acute infections and increased proportions of mice with chronic infections and decreased viral shedding. HTNV transmission risks and numbers of HFRS cases among civilian populations are greatly reduced from late December through early September the following year, most likely as a result of decreased viral shedding in chronically infected rodents. To support this, there are few reported HFRS cases among US military personnel reported from December to September the following year even though military exercises continue throughout the year, including when environmental conditions are conducive for increased production of dusts (dry season) and creating the potential for inhalation of dusts. To reduce HFRS risks, field training (especially involving rapid vehicular movements and artillery fires that produces excessive dusts) should be limited from late September-November at HTNV high-risk training sites. Rodent-borne disease surveillance should be conducted at military training sites to better understand rodent bionomics at different elevations and environments, including HTNV rodent-to-rodent transmission and viral shedding among acute and chronic cases, to assist in the development of comprehensive HFRS disease threats/ risks and to develop strategies to limit HTNV transmission risks to military and civilian populations.

### Hantavirus Ab+ Rates

Seasonal and annual hantavirus Ab+ rates were variable at lower elevation (<50 m) military training sites and were not correlated with increased numbers of hantavirus cases from September-December [5–8,27]. At NM-R, the highest hantavirus Ab+ rates were observed during the months preceding increased numbers of HFRS cases among Korean populations (KCDC 2012) and during periods of high gravid rates (April, August-September), suggesting increased transmission during the mating season [27,33]. The low/ moderate hantavirus Ab+ rates (range 2.1–14.3%) at NM-R compared to training sites at elevations <50 m near the DMZ may be the casual relationship of monthly trapping along the same trap lines that likely reduced rodent populations, removed hantavirus Ab+ rodents from the population, and reduced rodent-to-rodent transmission through wounding [30]. Thus, left undisturbed, hantavirus Ab+ rates may have exceeded 20% (considered to be high risk) during some periods.

Bank vole, *Myodes* (*Clethrionomys*) *glareolus* and primary reservoir of Puumala virus, populations in Europe vary annually with peak annual populations that correspond to peak numbers of human cases [31–32,34]. However *A*. *agrarius* populations (based on capture rates) are seasonally and annually similar, even following moderate (April) and high (August-September) reproductive periods [4–8,33]. Although HTNV Ab+ rates varied annually, when combined, overall seasonal HTNV Ab+ rates were not significantly different, indicating that chronic HTNV infections do not affect older populations. The hantavirus Ab+ *M*. *regulus* (1) and *M*. *minutus* (1) were likely due to interspecies competition as they were collected along the same trap lines as *A*. *agrarius*, while the Ab+ *C*. *lasiura* (1) was due to MJNV identified by PCR [13,35].

### Environmental Risks

Capture rates of *A*. *agrarius* exceeded 20% among tall grass habitats bordering dirt roads outside the primary maneuver and firing range and hardened and gravel roads leading to the main cantonment area. Tracked and wheeled vehicle speeds were limited due to the steep terrain and winding roads. The slow vehicular movement, in combination with low to moderate HTNV Ab+ rates, greatly reduces the potential for HTNV transmission to US military personnel conducting military operations. The margins of the modernized wheeled and tracked maneuver and firing range were not surveyed due to entry restrictions. However, available rodent habitat was limited as the vegetation along the moderately steep borders and within the maneuver area was cut short. The well maintained interior of the primary maneuver area likely harbored few *A*. *agrarius* and in addition to relatively slow vehicle movement reduced the potential for inhalation of dusts by vehicle occupants. While there have been documented cases of HFRS among US military personnel at military training sites <50 m, there have not been any documented cases attributed to exposure at NM-R over the past 20 years [3,TAK personal communication).

### Genomic Characterization

HTNV RNA gene fragments have been shown to vary geospatially [3]. In our phylogenetic results, the HTNV sequences obtained at NM-R (Pocheon) were closer with other HTNV sequences from *A*. *agrarius* captured from the Pocheon area (PC96-16), than other training sites, e.g. Firing Points 10 and 60, Yeoncheon; Twin Bridges Training Area (TBTA)-North and TBTA-South, Paju; and Dagmar North, Paju, which are 27.05, 24.25, 41.98, 43.29, 47.09 km distant, respectively, from NM-R. The HTNV sequences from NM-R form a distinct cluster compared to those from the other training sites. The characterization of HTNV RNA gene fragments provides a database based on geospatial variation of the HTNV genome that can be used to identify the source of HTNV infections when virus from HFRS patients and rodents captured from areas of exposure are characterized by RT-PCR. This is especially important due to the extended incubation period of up to 50 days and that many training exercises may be conducted at geospatially distant training sites over a period of one to several weeks. Taken together, and with the data presented here, field conditions during training that result in increased potential transmission can be determined and applied to strategies for disease mitigation based on training activities and seasonal periods of greater risks.

### Prevention

Surveillance of small mammals that identifies their relative abundance and hantavirus Ab+ rates, in addition to factors affecting transmission to soldiers while training in field environments, provides information that can be applied for disease threat analyses and the development of disease risk mitigation strategies. Thus, pre- and post-rodent-borne disease surveillance following interventions to limit exposure to rodent populations is necessary to identify their effect for mitigating transmission risks.

The etiology of HNTV is well known, with clinical infections that result in severe morbidity and mortality (5–10%) with the best of medical care among US personnel. Since MJNV was identified only recently and sera do not cross react with other rodent-borne hantaviruses, little is known about disease manifestation in humans, or if it does, the morbidity and mortality associated with infections. Limited numbers of *M*. *regulus* were collected and while one was hantavirus Ab+, Muju virus was not detected. The low numbers of *A*. *peninsulae*, often associated with higher elevations, and lack of detection of Soochong virus reduces the potential for its transmission at NM-R.

In summary, characterization of US and ROK operated training sites, in combination with small mammal bionomics, relative abundance, hantavirus Ab+ rates, and associated military training activities is essential for developing disease risk analyses and prevention. Therefore, long term rodent-borne disease surveillance should be an integral part of US military preventive medicine to improve our knowledge of military activities, environmental conditions, rodent bionomics, and seasonal periods that increase HTNV transmission risks in order to apply preventive medicine measures to mitigate disease risks.
